# Impact of Prophylactic Antibiotic Use in Ornamental Fish Tanks on Microbial Communities and Pathogen Selection in Carriage Water in Hong Kong Retail Shops

**DOI:** 10.3390/microorganisms12061184

**Published:** 2024-06-12

**Authors:** Chun Au-Yeung, Kit-Ling Lam, Man-Hay Choi, Ka-Wai Chan, Yu-Sum Cheung, Yat-Lai Tsui, Wing-Yin Mo

**Affiliations:** 1 Department of Applied Science, School of Science and Technology, Hong Kong Metropolitan University, Ho Man Tin, Kowloon, Hong Kong; 22123576r@connect.polyu.hk (C.A.-Y.); kllam@hkmu.edu.hk (K.-L.L.); mhchoi@hkmu.edu.hk (M.-H.C.); s1285279@live.hkmu.edu.hk (K.-W.C.); s1289007@live.hkmu.edu.hk (Y.-L.T.); 2 Department of Food Science and Nutrition, Faculty of Science, The Hong Kong Polytechnic University, Hung Hum, Kowloon, Hong Kong; yu-sum.cheung@polyu.edu.hk

**Keywords:** tetracyclines, fluoroquinolones, macrolides, PNEC, antibiotic resistance, zoonotic pathogens

## Abstract

Antibiotics are routinely added to ornamental fish tanks for treating bacterial infection or as a prophylactic measure. However, the overuse or subtherapeutical application of antibiotics could potentially facilitate the selection of antibiotic resistance in bacteria, yet no studies have investigated antibiotic use in the retail ornamental fish sector and its impact on microbial communities. The present study analyzed the concentrations of twenty antibiotics in the carriage water (which also originates from fish tanks in retail shops) collected monthly from ten local ornamental fish shops over a duration of three months. The antibiotic concentrations were correlated with the sequenced microbial community composition, and the risk of resistance selection in bacteria was assessed. Results revealed that the detected concentrations of tetracyclines were the highest among samples, followed by fluoroquinolones and macrolides. The concentrations of oxytetracycline (44.3 to 2,262,064.2 ng L^−1^) detected across three months demonstrated a high risk for resistance selection at most of the sampled shops. Zoonotic pathogens (species of *Rhodococcus*, *Legionella*, and *Citrobacter*) were positively correlated with the concentrations of oxytetracycline, tetracycline, chlortetracycline, and enrofloxacin. This suggests that antibiotic use in retail shops may increase the likelihood of selecting for zoonotic pathogens. These findings shed light on the potential for ornamental fish retail shops to create a favorable environment for the selection of pathogens with antibiotics, thereby highlighting the urgent need for enhanced antibiotic stewardship within the industry.

## 1. Introduction

Ornamental fish keeping is a popular hobby. The ornamental fish trade, a significant subsector of the aquaculture industry, is valued at up to USD 15–30 billion annually [1]. Ornamental fish are the third most popular pet choice, and approximately 14% of households in Hong Kong keep ornamental fish [2,3]. With the limited local supply, ornamental fish are often imported from different producing countries, and Hong Kong is ranked as the 14th largest importer in 2022 [4].

The Goldfish Market is a popular tourist destination as well as the largest ornamental fish retail area in Hong Kong. Aquarists can purchase prepackaged ornamental fish that are hung outside the shops or select their preferred individuals from the display tanks inside. Carriage water refers to the water used to transport ornamental fish, and it is usually taken from the fish tank housing the fish being transported. The stocking density of ornamental fish is extremely high, and it is not uncommon for a single ornamental fish shop to host more than several thousand ornamental fish. These conditions create a stressful environment for ornamental fish, potentially lowering their immunity and increasing their susceptibility to bacterial infection. For instance, streptococcosis and mycobacteriosis are common fish diseases that result from stress [5]. Retailers prioritize the survival of the fish before sale, as they do not exchange or refund once sold. The use of prophylactic antibiotics, which are antibiotics given to prevent bacterial diseases, has proven beneficial in maintaining the health of these fish, even under undesirable conditions in the display tanks [6].

The antibiotic use in the ornamental fish industry has led to the selection of antibiotic-resistant bacteria (ARB) and antibiotic resistance genes (ARGs) in the ornamental fish trade globally [7,8]. For instance, our previous study revealed that *Aeromonas* and *Pseudomonas* spp. isolated from the ornamental fish carriage water exhibited higher levels of minimum inhibitory concentrations (MICs) than those reported in the literature [9]. A similar finding by Verner-Jeffreys et al. suggested that nearly 50% of *Aeromonas* spp. isolated from carriage water samples demonstrated resistance to over 15 types of antibiotics, and most of them carried multiple ARGs, such as *qnrS2*, *intl1*, *tetD*, and *tetE* [6]. Moreover, it has been documented that antibiotics administered at subtherapeutic levels can induce the development of an environmental resistome [8,10]. For example, enrofloxacin at concentrations above 0.064 ng L^−1^ can result in the selection of ARB [11]. Therefore, antibiotic use in the ornamental fish industry should not be neglected, as it represents a potential source of ARB and ARGs that are closely linked to our daily lives. However, this issue has received much less attention than other aquaculture subsectors, as ornamental fish are not intended for human consumption.

Previous studies have primarily focused on the impact of antibiotic use on specific bacterial targets, leaving a gap in understanding of the impact on overall microbial communities. The present study determined the antibiotic residues and microbial community composition in carriage water, reflecting the history of drug application and the corresponding selection of ARB in ornamental fish tanks. The knowledge regarding the microbial communities associated with the prophylactic antibiotics in ornamental fish is rather limited, and the risk of antibiotics used in the ornamental fish industry on the selection of the environmental resistome has seldom been addressed. Next-generation high-throughput amplicon sequencing of the 16S ribosomal RNA (rRNA) gene is a powerful technique that can reveal microbial composition, diversity, and spatiotemporal patterns in the carriage water [12]. Compared to shotgun metagenomics, 16S rRNA gene sequencing offers key advantages for this study on water samples—it provides a cost-effective way to accurately quantify abundant bacterial communities that have a higher potential to reach disease-causing levels, and it can also effectively detect rare bacteria, including those at the genus level, while maintaining a lower rate of false positives [13,14]. The risk of antibiotic resistance selection in ornamental fish shops can be assessed using the risk quotient method by comparing the measured concentrations of antibiotic targets with the corresponding predicted no-effect concentration (PNEC) [15]. To our knowledge, this is the first study to characterize the microbial community composition in the carriage water of freshwater ornamental fish exposed to antibiotics. It also evaluates the risk of selecting ARB, providing valuable insights for both the industry and public health. Therefore, the objectives of this study were to (i) quantify the antibiotics used by various ornamental fish shops to understand their antibiotic consumption patterns; (ii) investigate the microbial communities and diversity in ornamental water tanks and their relationship with antibiotic use; and (iii) assess the risk of selecting ARB posed by the detected antibiotics in samples.

## 2. Materials and Methods

### 2.1. Research Premises and Sampling

During the sampling period, around 26 ornamental fish shops selling different types of fish were found in the Goldfish Market based on our preliminary survey, and slightly over 10 sold zebrafish (*Danio rerio*). Ten shops (S1–S10) that sold zebrafish with different fish stocking densities were selected, and this sample size should be representative. Zebrafish was selected in the present study, as this is one of the most popular pet fish [16]. The choice of a single species was made to avoid interspecies variations, as the microbial composition in carriage water could be affected by the fish species [12]. One pack of zebrafish was collected from each ornamental fish shop at a monthly interval over three consecutive months, and a total of 30 packs of zebrafish were collected from July to September 2023. Zebrafish were either purchased from shops with display tanks housing zebrafish, or prepacked zebrafish were chosen instead. Moreover, the sampling interval was applied to see the differences over time. The samples were placed in a shopper bag in which the inner layer was coated with aluminum foil, which can block sunlight to maintain the sampling condition for the samples. Samples were then transported to the laboratory immediately for further processing. The carriage water was filtered using a vacuum filtration system through a mixed cellulose ester filter (MCEF) membrane (0.2 µm, 47 mm, Millipore^TM^, Burlington, MA, USA). The membranes were then collected and stored at −80 °C until further processing, while the filtrate was used for antibiotic analyses.

### 2.2. Antibiotic Analyses

Twenty antibiotics, including tetracyclines (tetracycline (TC), chlortetracycline (CTC), oxytetracycline (OCT), and doxycycline (DC)), six fluoroquinolones (ciprofloxacin (CFX), enrofloxacin (EFX), ofloxacin (OFX), oxalinic acid (OA), sparfloxacin (SAR), and sarafloxacin (SFX)), three macrolides (clarithromycin (CTM), roxithromycin (RTM), and tylosin (TYL)), and six sulfonamides (sulfadiazine (SDZ), sulfamethazine (STZ), sulfamonomethoxine (SMM), sulfathiazole (SAZ), sulfamethoxazole (SMX), and sulfamerazine (SMZ)), were analyzed in carriage water samples. The specific antibiotics determined in the present study were those commonly utilized in both human health and husbandry practices, including aquaculture in China, and are often detected in various environmental settlings, including rivers and estuaries [17,18]. The extraction and quantification of these antibiotics were achieved using a solid phase extraction (SPE) procedure, followed by liquid chromatography electrospray ionization tandem mass spectrometry (LC-ESI-MS/MS) in positive ion mode [H^+^]. The methodology for antibiotic analysis was previously described in our earlier study [9]. Briefly, the filtered carriage water samples (300 mL) were spiked with 50 ppb of internal standards (sulfamethoxazole-13C6 (SMZ-13C6), ciprofloxacin-d8 (CFX-d8), roxithromycin-d7 (RTM-d7), and caffeine-13C3) after adjusting the pH to 3.5 using formic acid. The samples were passed through the Oasis HLB cartridge (Waters^®^, Milford, MA, USA) to remove interfering matrix constituents and concentrate the antibiotics for quantification. Each SPE cartridge was preconditioned with methanol, followed by ultra-pure water and acidified water. The samples were then loaded onto the SPE cartridge at a rate of 5 mL min^−1^. The antibiotics were eluted from the SPE cartridge using methanol and then concentrated to 0.2 mL under a gentle nitrogen stream. The extracts were subsequently reconstituted to a volume of 1 mL using a mixture of water and methanol in an 8:2 (*v*/*v*) ratio. The optimization conditions (retention time, precursor ions, product ions, and fragmentation) were detailed in our previous study [9].

The analysis was performed using an Agilent 1290 infinity LC system connected to an Agilent 6460 triple quadrupole mass spectrometer (Agilent, Palo Alto, CA, USA). Ten microliter of each sample were injected into an Agilent Infinity Lab Poroshell 120 EC-C18 (3.0 × 150 mm, 2.7 µm) with a guard filter. A binary mobile phase with gradient elution was employed for the analysis. Solvent A consisted of ultra-pure water containing 5 mM ammonium formate and 0.1% formic acid. Solvent B was a mixture of acetonitrile and methanol in an 80:20 ratio. The gradient started with 95% A for 2 min, followed by 60% A for 5 min, 55% A for 2 min, 40% A for 1 min, 95% A for 2 min, and held at 95% A for 0.5 min. The protonated ion ([M+H]^+^) was selected for the mass spectrometer analysis. The recoveries of the target compounds, determined by spiking two concentrations (50 and 100 ng L^−1^), were between 65 and 118% and 58.9 and 99.3%, respectively. The limits of quantification ranged from 0.03 to 0.95 ng L^−1^, while the limits of detection ranged from 0.01 to 0.31 ng L^−1^. The internal standard method was used to compensate for the matrix effects between the samples, and isotopically labeled standards were spiked in samples and calibration curve at the same concentration. The procedural blank was run to check for contamination, and a quality control sample was run every ten samples, followed by an injection of the solvent blank to ensure accuracy and precision. The details are provided in our previous study [9].

### 2.3. DNA Extraction

DNA extraction from MCEF membrane was performed using the NucleoMag DNA/RNA Water Kit (NucleoSpin^®^, Macherey-Nagel, Düren, Germany) in conjunction with a 5 mL bead tube (Type A 5 mL, Macherey-Nagel), following the manufacturer’s instructions. To prevent RNA contamination, RNase was added. Each extraction yielded 50 µL of DNA. The quality of the resulting DNA was subsequently assessed using a Nanodrop ND-1000 spectrophotometer (NanoDrop^®^ Technologies, Wilmington, DE, USA) and 1% gel electrophoresis. The DNA samples were then stored at −80 °C prior to sequencing.

### 2.4. Amplicon Sequencing and Bioinformatic Analysis

The DNA from each water sample was amplified at the V3–V4 region of the 16s rRNA gene with polymerase chain reaction (PCR) with primers 338F (5′-ACTCCTACGGGAGGCAGCAG-3′) and 806R (5′-GGACTACHVGGGTWTCTAAT-3′) (Beijing Genomics Institute, Hong Kong, China). The purified PCR products were used for library construction, followed by paired-end sequencing of the libraries on the DNBSEQ^TM^ platform with a 300 bp read length (Beijing Genomics Institute, Hong Kong, China). The resulting clean reads from raw data were clustered into operational taxonomic units (OTUs) with 97% similarity using USEARCH (v7.0.1090). These OTUs were then taxonomically classified using Ribosomal Database Project classifier software (v2.2) with a minimum confidence threshold of 0.6. The Greengenes database (V201305) was used for annotation, followed by calculation of species abundance at every taxonomic level, from phylum to species. Alpha diversity was estimated with MOTHUR software (version 1.31.2) and then analyzed based on the OTU table.

### 2.5. Risk Assessment of Resistance Selection

Risk assessment is a widely utilized tool for evaluating the impacts of anthropogenic pollutants, including antibiotics, on living organisms and ARB. This is achieved by comparing the concentration of a pollutant measured in the environment with its PNEC, which is derived by dividing the lowest observed adverse effect concentration or no observed effect concentration from toxicity studies by an assessment factor that accounts for uncertainties and extrapolations [15,16,19]. The risk of resistance selection posed by each detected antibiotic was calculated by the following formula:

RQrs = (MEC)/(PNECrs)

where RQrs = risk quotient for resistance selection; MEC = measured antibiotic concentration in the samples; and PNECrs = predicted no-effect concentration in the selection of ARB, which is adopted from Bengtsson-Palme and Larsson [11].

The PNECrs values showed the upper boundary for the minimal selective concentration of an antibiotic that can exert selective pressure at the community level. These values also suggest individual safety margins for antibiotics based on MICs data sourced from the database of the European Committee on Antimicrobial Susceptibility Testing (EUCAST). The risk was categorized into three levels: low (RQrs < 0.1); medium (0.1 ≤ RQrs < 1); and high (RQrs ≥ 1).

### 2.6. Data Analysis

The results of alpha diversity indices (observed species, Chao1, Shannon, and Simpson indices) were presented with their minimum, maximum, and mean values. One-way ANOVA followed by Dunnett post hoc test was applied in pairwise comparison. These statistical analyses were performed using Prism 9.4.1 for Mac (GraphPad Software, San Diego, CA, USA). Results are presented in graphs with corresponding *p*-values, and significant associations were determined by *p* < 0.05. Clustering, principal component analysis (PCA), and nonmetric multidimensional scaling (NMDS) ordination of antibiotics in carriage water were performed using PAST software (version 4.03). The redundancy analysis (RDA) to reveal the relationships between the antibiotic levels and microbial composition was also performed using PAST. Correlation analyses to reveal the relationships among antibiotic concentrations, alpha diversity, and the abundance of pathogenic genera were performed using Rstudio (v2023.12.1+402) using packages including corrplot. The correction was determined based on Pearson correlations at *p* < 0.05.

## 3. Results and Discussion

### 3.1. Antibiotic Concentrations

During the study period, 9 out of the 20 target antibiotics, including CTC, DC, OTC, TC, CFX, EFX, OA, CTM, and RTM, were detected in the carriage water samples. The mean concentrations of these antibiotics are shown in Table 1, and the detailed detected concentrations of individual sample are summarized in Table S1. These antibiotics belong to three classes, including tetracyclines, fluoroquinolones, and macrolides. Notably, no sulfonamide antibiotics were detected in the samples. The total detected antibiotic concentrations ranged from 95.0 to 2,472,043.7 ng L^−1^. Shop S1 had the lowest total antibiotic concentration, while shop S3 had the highest. The total concentrations of tetracyclines, fluoroquinolones, and macrolides ranged from 81.0 ng L^−1^ to 2,471,912.7 ng L^−1^, 8.0 ng L^−1^–126.5 ng L^−1^, and 1.2 ng L^−1^–19.2 ng L^−1^, respectively. All samples, with the exception of CTC in shop S5, had tetracyclines throughout the study period. OTC exhibited the highest concentration among all tetracyclines, ranging from 44.3 to 2,262,064.2 ng L^−1^. For fluoroquinolones, CFX and EFX were detected in almost all samples, with concentrations ranging from 1.7 to 403.7 ng L^−1^ and 2.4 to 124.7 ng L^−1^, respectively. Among all the macrolides, RTM exhibited the highest concentration, varying between 1.1 and 19.1 ng L^−1^. These findings suggested that tetracyclines was the most commonly used antibiotic class in ornamental fish shops compared to the other three antibiotic classes. These findings were consistent with our previous findings and other similar research [9,20]. Tetracyclines is the most popular antibiotic class used for ornamental fish, particularly oxytetracycline [21]. However, it has been reported that the microbial communities and metabolic pathways of zebrafish were altered when they were exposed to high concentrations of OTC (1000 and 5000 ng mL^−1^), including an increase in the abundance of pathogenic bacteria [22]. Given that the antibiotic residue in the fish tank can end up in the environment, the presence of antibiotics in water bodies might also have acute and chronic toxic effects on other aquatic organisms, such as algae and invertebrates [23,24]. For example, *Microcystis aeruginosa* was very sensitive to OTC, and the EC_50_ is 0.207 mg L^−1^ [25]. In addition to ecological implications, tetracyclines also pose health risks to human such as skin pigmentation and irritation, bone and teeth damage, and lupus erythematosus [26]. Tetracyclines can inhibit a wide range of Gram-positive and Gram-negative bacteria and atypical organisms such as mycoplasmas and protozoan parasites [27,28]. Infections caused by bacteria and parasites are common in ornamental fish, often resulting from environmental conditions and stress caused by high stocking density and poor water quality during production or transportation. Additionally, antibiotics are commonly administered through baths, in-feed, gavage, and injection [21]. OTC baths have been reported to be effective and are extensively used in bacterial infections such as aeromonosis, pseudomonosis, and vibriosis in fish [9,29,30]. Therefore, the use of tetracyclines in ornamental fish shops has become a standard practice to prevent infections.

### 3.2. Cluster Analysis and Principal Component Analysis of Antibiotics in Fish Carriage Water

**Figure 1:**
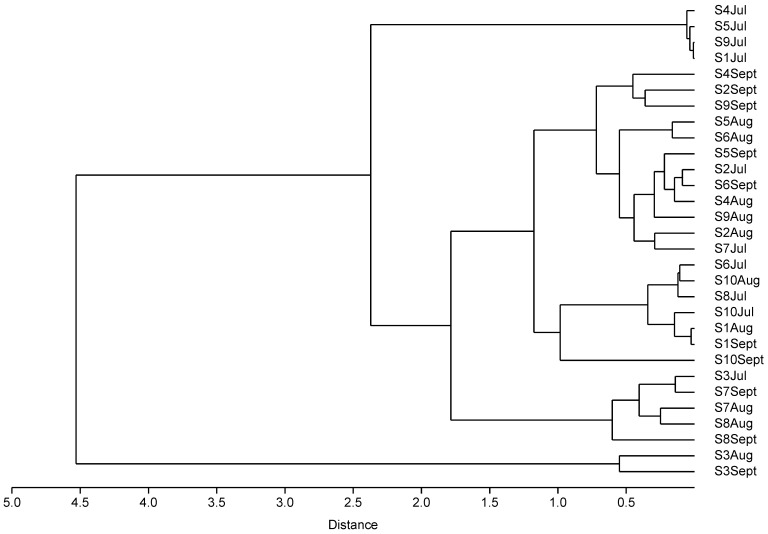
Cluster analysis dendrogram of antibiotics detected in carriage water samples.

To investigate the usage patterns of antibiotics among the ornamental fish shops in Hong Kong, cluster analysis was conducted using the 30 samples collected (Figure 1). Clusters were formed according to the detected antibiotic concentrations and types rather than shops and time. Group 1 contained samples collected from shop S3 in Aug and Sept, which exhibited the highest concentrations of TC and OTC. Group 2 included samples collected from shops S1, S4, S5, and S9 in Jul, which displayed the lowest antibiotic concentrations. Group 3 consisted of samples collected from shop S3 in Jul, shop S7 in Aug and Sept, and shop S8 in Aug, which showed relatively high concentrations of TC and OTC. These results indicate variations in antibiotic usage across different shops and time periods. Recommended OTC bath usage is 1 h at 20 mg L^−1^ up to 4 days, with withdrawal periods of 21–60 days for investigational use in fish [31]. In our finding, continuous uses of antibiotics were observed, and the use of certain antibiotics also increased significantly month after month. For example, the concentrations of total tetracyclines in shops S2 and S6 doubled every month, and 100-fold increases in the total tetracyclines concentrations were observed in shops S3 and S8 (Table S1). On the other hand, the variations in total concentrations of fluoroquinolones and macrolides were not significant across the sampling period. These results indicated variations in antibiotic usage practices across different shops and time periods, with a continuous use of OTC and TC but more random use for fluoroquinolones and macrolides. The antibiotic usage patterns seem to depend on the specific types of antibiotics.

**Figure 2:**
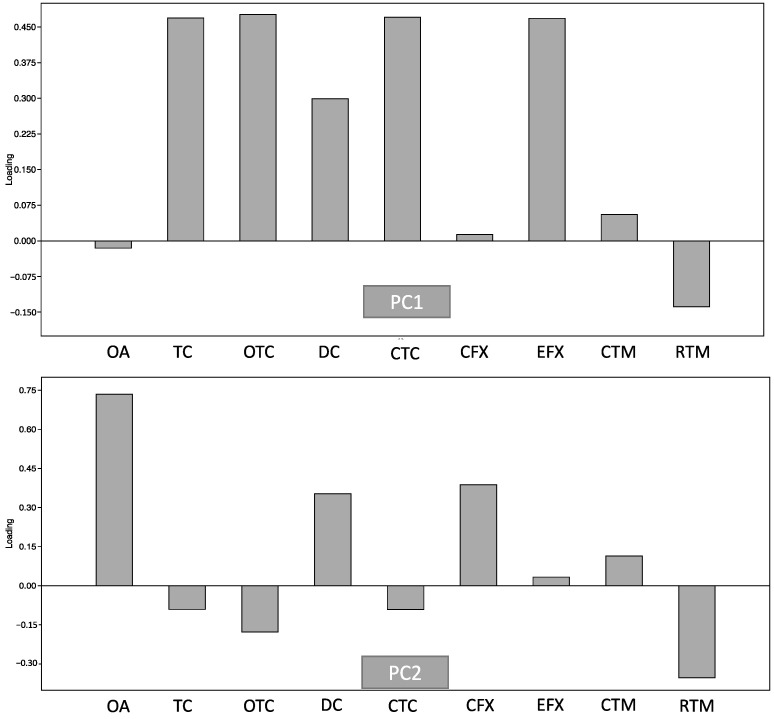
Principal component analysis of antibiotics detected in carriage water samples.

PCA was further applied to describe the usage patterns of antibiotics in ornamental fish shops (Figure 2). PC1 and PC2 explained about 99.0% of the variance in antibiotic usage, with PC1 accounting for 97.5% and PC2 for 2.1% of the explained variance, respectively. The most influential factors in PC1 were TC, OTC, CTC, and EFX, indicting a dominance of the use of tetracycline antibiotics. PC2 was mainly influenced by OA, DC, and CFX. These confirmed tetracyclines (TC, OTC, and CTC) were primarily used in ornamental fish shops. Although CTC, OTC, and TC are often used in food animals such as fish, bovine, and poultry, tetracyclines are still considered clinically relevant groups of antibiotics for many infectious diseases, such as respiratory infection and malaria [28,32]. Therefore, high concentrations of tetracyclines in ornamental fish shops can endanger human health by facilitating the emergence of ARB, reducing drug effectiveness, and posing toxicity risks to other animals in the environment [28,32]. However, in ornamental fish, antibiotic regulation is more limited than in agricultural animals and humans.

### 3.3. The Composition and Diversity Analysis of Microbial Community

#### 3.3.1. Microbial Composition and Structure among the Carriage Water Samples

**Figure 3:**
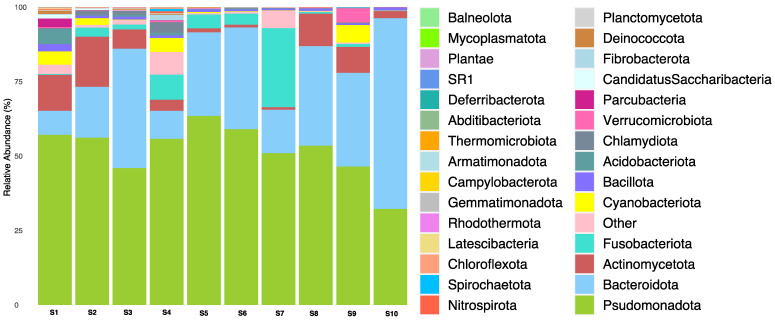
Relative abundance of bacterial phylum in carriage water samples.

A total of 4345 OTUs were classified into seven taxonomic orders, ranging from the phylum to the species level. The microbial composition of the phylum is depicted in Figure 3. The most abundant phyla across the carriage water samples were Pseudomonadota (32.2–63.5%), followed by Bacteroidota (8.1–64.1%), Fusobacteriota (0.1–26.5%), and Actinomycetota (0.8–16.8%). However, the microbial composition in the carriage water varied between shops. For example, Bacteroidota dominated in shop S10, but was much less prevalent in shops S1, S4, and S7. Similarly, the abundance of Fusobacteriota in shop S7 was at least three times higher than that in other shops. Moreover, the abundance of Actinomycetota was lower in shops S5–S7 compared to the others. On the other hand, fewer common phyla such as Chloroflexota, Nitrospirota, and Spirochaetota were observed in other samples, suggesting the higher microbial complexity within these samples. The dominant phyla identified in the carriage water samples were Pseudomonadota, Bacteroidota, Fusobacteriota, and Actinomycetota, which were similarly found in ornamental fish aquarium water in the United States [12].

**Figure 4:**
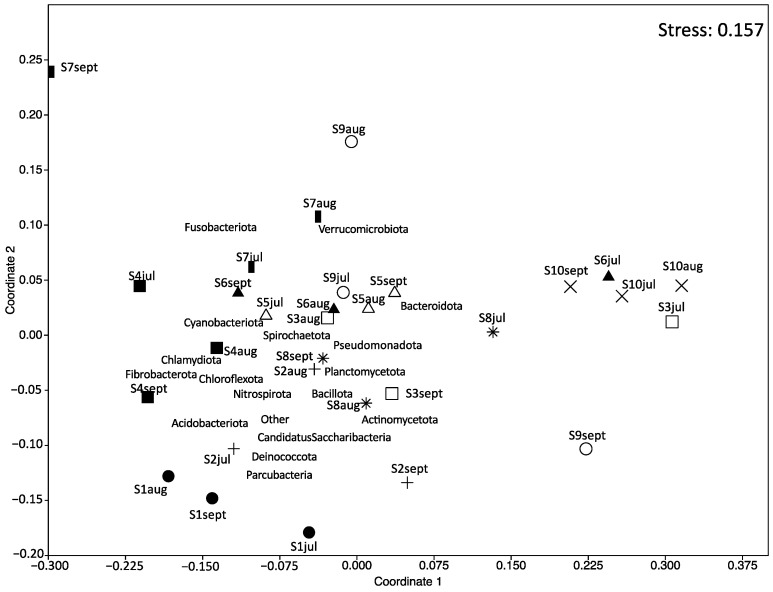
Nonmetric multidimensional scaling (NMDS) ordination plot of the microbial composition of carriage water samples. Different symbols represent different shops: fill-circle = S1; plus = S2; square = S3; fill-square = S4; triangle = S5; fill-triangle = S6; bar = S7; star = S8; circle = S9; cross = S10.

NMDS ordination was applied to demonstrate the similarity of microbial composition between the samples (Figure 4). The results showed that carriage water samples collected from shops S2, S3, S6, S7, S8, and S9 at different months were farther apart from each other, implying a change in the microbial composition over time within these ornamental fish shops. However, the microbial composition of samples collected from shops S1, S4, S5, and S10 clustered relatively close together, suggesting that their microbial compositions remained relatively stable over time in these shops.

#### 3.3.2. Alpha Diversity of Microbial Communities

**Figure 5:**
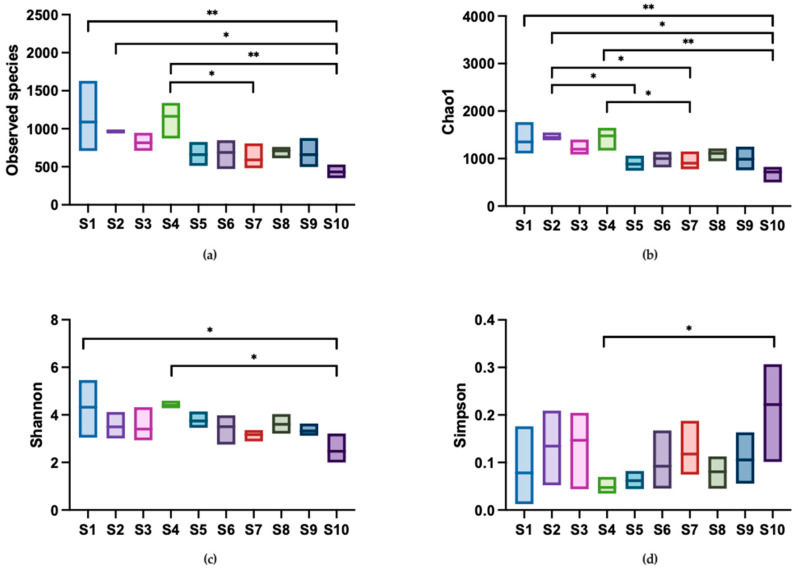
Comparison of alpha diversity indices among sampling shops. Diversity in the microbial community of ornamental fish carriage water was measured using (**a**) observed species, (**b**) Chao1, (**c**) Shannon, and (**d**) Simpson index. Asterisks indicate statistically significant differences in pairwise comparisons (* *p* < 0.05 and ** *p* < 0.01) (*n* = 3).

Alpha diversity indices, including observed species, Chao1, Shannon, and Simpson indices, are shown in Figure 5. The observed species and Chao1 indices reflect the community richness in samples. The observed species index ranged from 349 to 1631, with shop S1 exhibiting the highest unique OTU present in the water and shop S10 exhibiting the lowest unique OTU. A similar pattern was observed in the Chao1 index. In terms of community diversity, the Shannon index ranged from 1.99 to 5.43, while the Simpson index, which inversely correlates with diversity (a lower index indicates higher diversity), ranged from 0.04 to 0.31. These results suggested that the carriage water collected from ornamental fish shops had diverse microbial communities. In terms of variation in alpha diversity across the shops, community richness was higher than community diversity. This was evident as the number of significant differences observed in the observed species and Chao1 indices were greater than those in the Shannon and Simpson indices. These findings indicated distinct differences in community richness across the shops, but a relatively similar community diversity.

#### 3.3.3. Pathogenic Bacteria Presented in Carriage Water Samples

**Figure 6:**
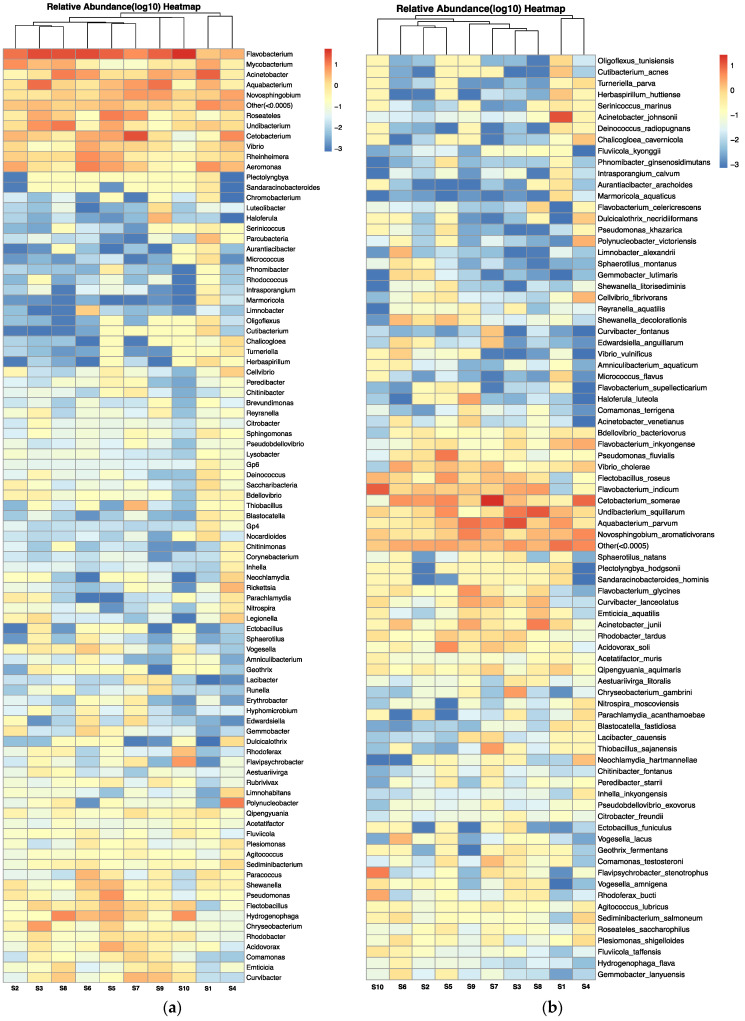
Heatmap of log10 relative abundance of microbial composition at (**a**) genus level and (**b**) species level (relative abundance > 0.0005%) in carriage water samples.

The microbial composition of the carriage water, at both genus and species level (relative abundance > 0.0005%), is depicted in Figure 6a,b, respectively, using a heatmap of log_10_ relative abundance. Pathogenic genera and species refer to those that are capable of causing harm to either humans or aquaculture species (Supplementary Table S2). In total, 13 pathogenic genera (out of 91) and 18 pathogenic species (out of 82) were identified in the carriage water samples. Certain genera, including *Flavobacteria*, *Mycobacterium*, *Acinetobacter*, *Vibrio*, and *Aeromonas*, were predominantly found across samples, with a relative abundance close to 10% (Figure 6a). Several genera of bacteria with zoonotic airborne potential were found in the current study, including species of *Mycobacteria*, *Vibrio*, and *Pseudomonas*. In addition, *Legionella* spp. was detected in samples collected from shops S1, S2, S3, and S4. At the species level (Figure 6b), several pathogens known to be harmful to humans were also identified in the samples, such as *Vibrio cholera*, *Vibrio vulnificus*, *Citrobacter freundii*, *Acinetobacter johnsonii*, *Blastocatella fastidiosa*, and *Plesiomonas shigelloides*. Furthermore, certain bacterial species could result in serious diseases in humans, such as nontuberculous mycobacterial infections caused by the *Mycobacterium avium* complex group, Legionnaires’ disease caused by *Legionella* spp., pneumonia infection caused by *Parachlamydia acanthamoeba*, and multidrug-resistant infection caused by *Pseudomonas aeroginosa*, which were reported to be resistant to various antibiotics [33,34,35,36].

As this is the first study on the effects of antibiotics on microbial structure in carriage water originating from retail fish shops, it is impossible to compare with similar retail environments in other parts of the world. Nevertheless, an analysis of the microbial structure in carriage water from U.S. pet stores revealed that the pathogenic genera isolated from carriage water were similar to those found in the present study. The pathogen genera *Vibrio*, *Legionella*, *Mycobacterium*, and *Aeromonas* were commonly prevalent in carriage water in U.S. retail stores as well [12]. The release of pathogenic bacteria from stressed or deceased fish is a common concern in the ornamental fish industry. The phenomenon can lead to an increased risk of disease transmission. Therefore, it can be reasonably inferred that fish tanks in retail shops act as incubation sites for both human and animal pathogens, potentially posing a health risk to employees and customers. Furthermore, the presence of these pathogenic bacteria in conjunction with prophylactic antibiotics suggests a probable development of multidrug resistance. Exposure to these resistant pathogens, which possess zoonotic potential, could pose a significant threat to human health.

### 3.4. Multivariate Correlation Analysis between Microbial Composition at Phylum Level and Antibiotics

**Figure 7:**
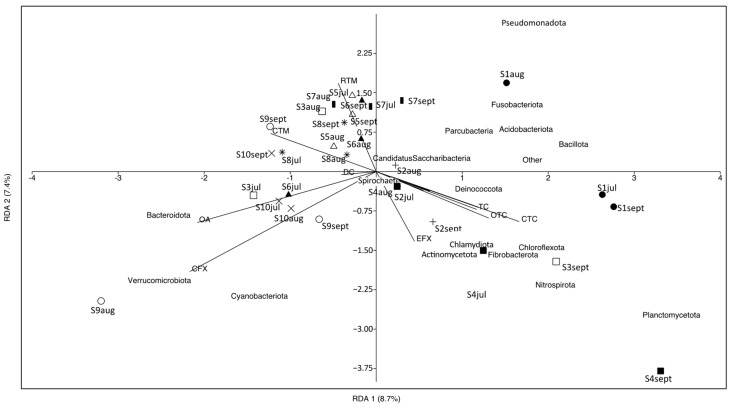
Redundancy analysis of microbial communities at phylum level of carriage water and antibiotic concentrations. Different symbols represent different shops: fill-circle = S1; plus = S2; square = S3; fill-square = S4; triangle = S5; fill-triangle = S6; bar = S7; star = S8; circle = S9; cross = S10.

RDA was performed to determine the correlation between antibiotics and the microbial communities at the phylum level in carriage water samples (Figure 7). Nine detected antibiotics accounted for 30% of the total variation in microbial communities. The first two canonical axes explained 8.7% and 7.4% of the total variation, respectively, indicating that the use of antibiotics in carriage water samples could only explain a small proportion of the variation in microbial communities. It is important to note that microbial communities in the carriage water can be affected by various factors, such as the source of the ornamental fish, the maintenance routine adopted by different shops, the water quality of fish tanks, and the fish types sold. A previous study reported significant differences in microbial communities in carriage water associated with different fish types [11]. While our study sampled the same fish species, it remains challenging to completely eliminate the effects of fish species on microbial communities. Some fish shops utilize centralized filtration systems to host different fish species, which could introduce cross-contamination. Moreover, ornamental fish sold in different shops might originate from different sources, and microbial communities acquired by fish during their initial rearing could also affect the microbial composition in the carriage water within the fish shops.

A limitation of the present study is the absence of data on microbial communities associated with the ornamental fish themselves before they were introduced into the fish tanks, and data on microbial communities in other fish tanks. Additionally, it was difficult to analyze the microbial communities in other fish tanks or across different ornamental fish species sold within the same shop. To provide a more comprehensive understanding of the factors shaping the microbial community in ornamental fish tanks, future studies should consider incorporating factors such as fish source, maintenance practices, water quality, and fish species diversity when investigating microbial communities in carriage water associated with ornamental fish shops. This will provide a more holistic understanding of the influences on the microbial community in ornamental fish tanks.

### 3.5. Correlation between Antibiotic Concentration, Alpha Diversity Indices, and Pathogen Genera

**Figure 8:**
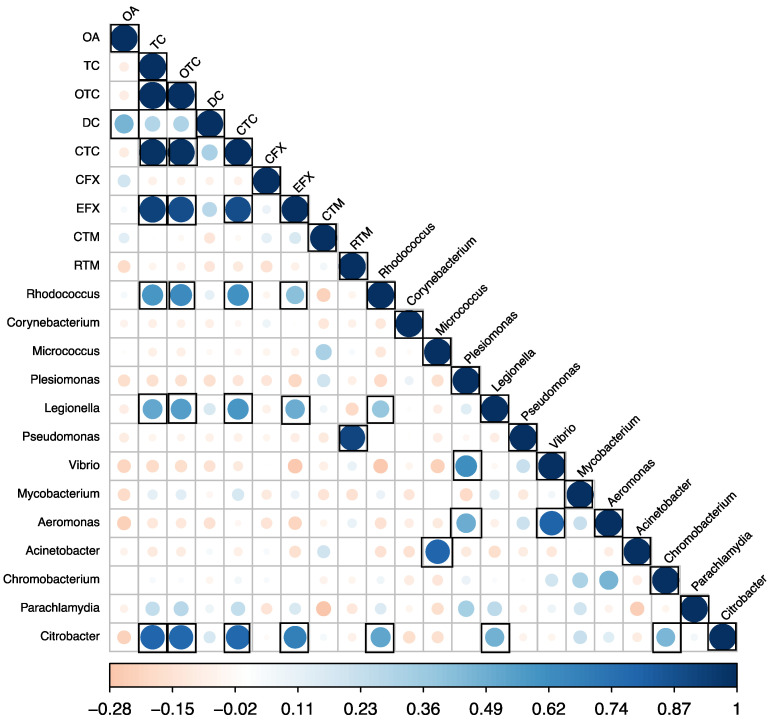
Pearson correlations between antibiotic concentrations and the abundance of pathogen genera in samples (*n* = 30). Black boxes indicate a significant value at *p* < 0.05.

Pearson correlations were calculated between the detected antibiotic concentrations, alpha diversity indices, and abundance of pathogenic genera. No significant correlations were found between the detected antibiotics and alpha diversity indices. However, significant positive correlations (*p* < 0.05) were observed between the detected antibiotic concentrations in carriage water and the abundance of pathogen genera (Figure 8). Specifically, tetracycline (TC), oxytetracycline (OTC), chlortetracycline (CTC), and enrofloxacin (EFX) positively correlated with species of *Rhodococcus* (r = 0.58, 0.63, 0.61, and 0.42, respectively), *Legionella* (r = 0.52, 0.56, 0.59, and 0.49, respectively), and *Citrobacter* (r = 0.80, 0.79, 0.79, and 0.69, respectively). Previous studies reported that tetracyclines are a broad-spectrum group of antibiotics that demonstrate activity against a wide range of both Gram-positive and Gram-negative bacteria [37,38]. In contrast to other antibiotic classes, tetracyclines exert their antimicrobial effects by inhibiting protein synthesis in susceptible bacteria by binding to the 30S ribosomal subunits to prevent proper protein synthesis [38]. Additionally, tetracyclines can also interact with the 70S ribosomes found in bacterial mitochondria [38]. Through these mechanisms of action, tetracycline antibiotics exhibit potent efficacy against actively multiplying bacteria. RTM was also found to positively correlate with the abundance of *Pseudomonas* spp. (r = 0.91). These findings suggested that while antibiotic usage in tank water or carriage water did not impact microbial diversity, it contributed to an increased abundance of pathogenic genera.

High levels of antibiotics were found in the carriage water, but a large variety of microorganisms was detected, particularly the Gram-negative Pseudomonadota. On the other hand, Gram-positive bacteria, such as *Streptococcus*, *Lactococcus*, *Enterococcus*, and *Vagococcus*, which are commonly found in fish tanks [39,40,41], were not observed in our 16S sequencing results. These results implied that the antibiotics used could effectively inhibit or eliminate certain Gram-positive bacteria, but also favored the selection of Gram-negative bacteria. The presence of pathogenic bacteria along with high levels of antibiotics in the carriage water suggested that the bacteria, particularly the Gram-negative ones, may have developed resistance to antibiotics. Liu et al. reported that ornamental fish markets could be an unperceived reservoir for the emergence and dissemination of ARGs, as evidenced by observed positive correlations between antibiotic concentrations and ARGs in water bodies [17]. Considering that high levels of antibiotics contribute to the development of resistance in bacteria, our results suggested that the application of high concentrations of antibiotics in ornamental fish shops favored the selection of ARB [42]. It is necessary to raise the awareness in fish shop workers, government officials, and the public of the problem of antibiotic-resistant bacteria and reduction of the use of antibiotics.

Among the ten sampled shops, shop S1 had both the lowest stocking density and the lowest detected antibiotic levels, suggesting that a lower fish density per tank could reduce the need for antibiotics. Thus, the fish shop workers should avoid using antibiotics and employ alternative methods such as reducing stocking density, monitoring water-quality of fish tanks, or applying herbal Chinese medicine to minimize stress, thereby improving the immunity and healthiness of the fish [43,44]. Moreover, the use of ozone nanobubbles could be an effective means to control the nontuberculous *Mycobacterium* spp. in water bodies [45].

Furthermore, species of *Rhodococcus*, *Legionella*, and *Citrobacter* that can cause pneumonia, especially in vulnerable groups like children and the elderly [46,47,48], were detected. For example, *Legionella pneumophila* has been linked to sporadic outbreaks of pneumonia. Notably, these three bacterial genera may also be airborne pathogens. Blatny et al. (2008) observed that *Legionella* spp. were detected up to 200 m downwind from the aeration ponds of a sewage treatment plant [49]. Moreover, species of *Citrobacter* and *Rhodococcus* have been isolated in aerosol samples from various environments [50,51]. These findings suggest that the use of antibiotics in ornamental fish shops could contribute to the selection of airborne pneumonia-related pathogens. The selection of zoonotic pathogens in the fish tanks of ornamental fish shops could also pose occupational health and safety risks to the shopkeepers, as they spend much longer periods in the shops and could have a higher chance of contacting or breathing in those bacteria. Consequently, workers, children, and the elderly visiting the shops may be at risk, and it will be important to quantify the relevant risk.

### 3.6. Risk Assessment of Resistance Selection

The RQrs were estimated to assess the risk of resistance selection posed by the antibiotics detected in the carriage water, and the results are shown in Table 2. The risk of resistance selection in carriage water samples collected from local ornamental fish shops varied from low to high. The RQrs for the detected concentrations of TC in shops S3, S7, and S8, CFX in S9, and EFX in S3 were 208.7, 1.2, 1.4, 6.3, and 1.9, respectively, indicating a high risk of resistance selection. Additionally, the RQrs of OTC in all samples, except S2, were at least 21 times higher than the risks posed by TC, CFX, and EFX, ranging from 2.1 to 4524.1. In contrast, the remaining detected antibiotics demonstrated medium to low risks. These findings suggested that OTC posed the greatest concern. In the present study, we observed that OTC exhibited the highest concentrations among all carriage water samples, reaching up to 2 mg L^−1^. Moreover, its concentration showed a positive correlation with the abundance of species of *Rhodococcus*, *Legionella*, and *Citrobacter*, further confirming OTC’s potential risk in selecting resistant pathogens.

OTC is still widely used as a first-line antibiotic treatment for many diseases, such as Legionnaires’ disease, traveler’s diarrhea, Coxiellosis, and Brucellosis (zoonotic bacterial infection diseases) [52,53]. The use of OTC in ornamental fish shops can potentially contribute to the shortage of effective therapies for bacterial infections. We believe that the display tanks of ornamental fish shops could serve as incubators for tetracycline-resistant pathogens due to the administration of high concentrations of OTC. The purchase of ornamental fish from retail shops and their introduction into home fish tanks could inadvertently spread these pathogens and their resistance genes, posing significant health risks to immunocompromised individuals as well as occupational safety concerns for shop workers. Therefore, we recommend monitoring the use of antibiotics in ornamental fish shops, similar to the regulations applied in the food production sector.

## 4. Conclusions

This study provides novel insights into the relationships between the use of antibiotics and the microbial composition in ornamental fish shops. Consistent with our previous study, extensive use of OTC and TC (tetracycline) was observed throughout the study period, with particularly high levels of OTC. The concentrations of other antibiotics like CTC, DC, CFX, EFX, OA, CTM, and RTM were below therapeutic levels. Analysis of the sequenced microbial community identified potential zoonotic pathogens, including species of *Rhodococcus*, *Legionella*, and *Citrobacter*, which positively correlated with OTC, TC, CTC, and EFX levels. This suggests that these four antibiotics may pose high risks for selecting potential zoonotic pathogens. Among all detected antibiotics, the use of OTC in ornamental fish shops warrants special attention, as it exhibited the highest concentrations and posed the highest risk of resistance selection. Furthermore, OTC is a first-line treatment for many diseases. To protect public health and preserve effective therapies, we recommend monitoring and regulating the use of antibiotics in ornamental fish shops. Overall, this study confirmed the widespread application of antibiotics in ornamental fish shops and highlighted the associated risks of selecting antibiotic-resistant pathogens. These findings emphasize the need for comprehensive studies and improved antibiotic management practices to safeguard workers and the public health by promoting more judicious antibiotic use in ornamental fish shops.

## Figures and Tables

**Table 1:** Antibiotic concentrations detected in the carriage water samples.

Carriage Water from Shops	Concentration (ng L^−1^) of Antibiotic												
	CTC	DC	OTC	TC	Subtotal of TCs	CFX	EFX	OA	Subtotal of FQs	CTM	RTM	Subtotal of MLs	Total
S1	12.9	13.3	44.3	10.6	81.0	4.4	6.1	N.D.	10.5	0.1	3.4	3.5	95.0
S2	14.8	21.5	19,427.5	239.1	19,703.0	7.3	19.9	N.D.	27.2	0.1	3.1	3.2	19,733.5
S3	144.9	971.7	2,262,064.2	208,732.0	2,471,912.7	1.7	124.7	N.D.	126.5	0.1	4.4	4.5	2,472,043.7
S4	11.8	16.6	9762.2	87.0	9877.6	17.1	26.9	N.D.	44.0	0.1	1.1	1.2	9922.8
S5	N.D.	13.1	5237.5	48.5	5299.2	2.4	10.9	N.D.	13.3	0.1	19.1	19.2	5331.7
S6	7.9	13.6	3453.4	54.8	3529.8	8.7	2.4	N.D.	11.2	0.1	3.5	3.6	3544.5
S7	5.8	36.0	31,355.8	1229.3	32,627.0	8.0	N.D.	N.D.	8.0	0.1	1.2	1.3	32,636.3
S8	N.D.	57.0	65,440.9	1372.7	66,870.5	N.D.	8.7	N.D.	8.7	0.1	3.5	3.6	66,882.8
S9	3.9	18.6	9073.0	141.1	9236.6	403.7	25.6	21.5	450.8	0.1	1.1	1.2	9688.6
S10	4.3	13.6	1074.6	31.3	1123.8	4.7	26.4	90.0	121.2	0.1	1.1	1.2	1246.2

N.D. = not detected; values are represented as mean (*n* = 3).

**Table 2:** Risk quotients for antibiotics detected in carriage water originating from the ten sampled ornamental fish shops.

Fish Shops	Risk Quotients								
	CTC	DC	OTC	TC	CFX	EFX	OA	CTM	RTM
S1	N.A.	0.007	0.09	0.01	0.07	0.09	N.A.	0.0005	0.003
S2	N.A.	0.01	38.9	0.2	0.1	0.3	N.A.	0.0005	0.003
S3	N.A.	0.5	4524.1	208.7	0.03	1.9	N.A.	0.0005	0.004
S4	N.A.	0.008	19.5	0.09	0.3	0.4	N.A.	0.0005	0.001
S5	N.A.	0.007	10.5	0.05	0.04	0.2	N.A.	0.0005	0.02
S6	N.A.	0.007	6.9	0.05	0.1	0.04	N.A.	0.0005	0.003
S7	N.A.	0.02	62.7	1.2	0.1	0.00001	N.A.	0.0006	0.001
S8	N.A.	0.03	130.9	1.4	0.000001	0.1	N.A.	0.0005	0.003
S9	N.A.	0.009	18.1	0.1	6.3	0.4	N.A.	0.0005	0.001
S10	N.A.	0.007	2.1	0.03	0.07	0.4	N.A.	0.0005	0.001
PNECrs (ng L^−1^) ^1^	N.A.	2000	500	1000	64	64	N.A.	250	1000

^1^ PNECrs, predicted no-effect concentration for resistance selection; the PNEC corresponds to the size-adjusted lowest MIC as reported by Bengtsson-Palme and Larsson [11]; N.A., not applicable. The risk was categorized into three levels: low (RQrs < 0.1); medium (0.1 ≤ RQrs < 1); and high (RQrs ≥ 1).

## Data Availability

The original contributions presented in the study are included in the article/Supplementary Materials, further inquiries can be directed to the corresponding author.

## Supplementary Materials

The following supporting information can be downloaded at: https://www.mdpi.com/article/10.3390/microorganisms12061184/s1, Table S1: The summary of detected antibiotic concentrations of each samples (*n* = 30, ng L^−1^); Table S2: Potential pathogenic bacteria detected in samples. References [54,55,56,57,58,59,60,61,62,63,64,65,66,67,68,69,70] are cited in the Supplementary Materials.
