# Supplementary material for: Impact of Prophylactic Antibiotic Use in Ornamental Fish Tanks on Microbial Communities and Pathogen Selection in Carriage Water in Hong Kong Retail Shops
Source: Microorganisms. 2024 Jun 12;12(6):1184. doi: 10.3390/microorganisms12061184 (PMC11205468; doi:10.3390/microorganisms12061184)
Supplement: Supplementary file 1 [file microorganisms-12-01184-s001.zip › microorganisms-3014223-supplementary.pdf]

Table S1. The summary of detected antibiotic concentrations of each samples (n = 30, ng L^{-1}).

Shop	Sample name	CTC	DC	OTC	TC	Subtotal of TCs	CFX	EFX	OA	Subtotal of FQs	CTM	RTM	Subtotal of MLs	Total
S1	S1jul	N.D	8.3	29.4	0.4	38.0	4.1	8.6	N.D	12.7	N.D	4.3	4.3	55.0
	S1aug	18.8	20.1	33.7	15550.0	15622.5	8.8	7.9	N.D	16.7	0.4	6.0	6.3	15645.5
	S1sept	19.9	11.4	69.8	16227.9	16329.0	0.5	1.6	N.D	2.1	N.D	N.D	0.0	16331.1
S2	S2jul	0.0	11.0	6118.5	96359.1	102488.5	4.8	10.7	N.D	15.5	N.D	3.3	3.3	102507.3
	S2aug	19.8	20.8	12364.3	216545.8	228950.7	12.6	27.3	N.D	39.9	0.4	6.0	6.4	228997.0
	S2sept	24.6	32.9	39799.8	404523.3	444380.6	4.6	21.7	N.D	26.3	N.D	N.D	0.0	444406.8
S3	S3jul	N.D	10.8	51807.5	2717402.0	2769220.3	4.2	17.0	N.D	21.2	N.D	5.9	5.9	2769247.5
	S3aug	125.6	779.8	1884959.1	220373330.6	222259195.1	0.0	171.4	N.D	171.4	0.4	7.1	7.5	222259374.0
	S3sept	309.0	2124.4	4849426.0	403105237.1	407957096.5	1.0	185.7	N.D	186.8	N.D	N.D	0.0	407957283.3
S4	S4jul	N.D	8.2	200.1	0.4	208.7	5.1	12.6	N.D	17.7	N.D	3.3	3.3	229.6
	S4aug	16.7	20.1	7373.9	124377.8	131788.4	30.6	38.9	N.D	69.5	0.4	N.D	0.4	131858.3
	S4sept	18.7	21.5	21712.7	136670.5	158423.4	15.7	29.1	N.D	44.9	0.0	N.D	0.0	158468.3
S5	S5jul	N.D	7.9	108.5	0.4	116.8	3.4	8.1	N.D	11.5	N.D	39.2	39.2	167.5
	S5aug	N.D	13.9	3955.7	55643.8	59613.5	N.D	16.2	N.D	16.2	0.4	14.2	14.5	59644.2
	S5sept	N.D	17.5	11648.4	89810.7	101476.6	3.8	8.5	N.D	12.3	N.D	3.9	3.9	101492.7
S6	S6jul	N.D	7.4	523.5	27066.5	27597.5	3.5	7.3	N.D	10.8	N.D	3.4	3.4	27611.6
	S6aug	N.D	15.5	2446.3	51424.4	53886.2	9.1	N.D	N.D	9.1	0.4	7.1	7.5	53902.7
	S6sept	23.8	17.9	7390.5	85889.4	93321.5	13.6	N.D	N.D	13.6	N.D	N.D	0.0	93335.1
S7	S7jul	N.D	11.5	6577.1	308800.8	315389.4	0.0	N.D	N.D	0.0	N.D	3.5	3.5	315392.9
	S7aug	N.D	48.4	23476.6	1242206.8	1265731.7	7.8	N.D	N.D	7.8	0.4	N.D	0.4	1265740.0
	S7sept	17.5	48.3	64013.8	2136932.6	2201012.1	16.3	N.D	N.D	16.3	N.D	N.D	0.0	2201028.4
S8	S8jul	N.D	7.8	993.4	31070.0	32071.2	0.0	12.4	N.D	12.4	N.D	3.3	3.3	32086.9
	S8aug	N.D	50.8	42291.4	1221786.3	1264128.5	0.0	9.6	N.D	9.6	0.4	7.2	7.6	1264145.6
	S8sept	N.D	112.5	153037.8	2865213.6	3018363.8	0.0	4.0	N.D	4.0	N.D	N.D	0.0	3018367.8
S9	S9jul	N.D	8.2	44.8	0.4	53.4	9.7	N.D	N.D	9.7	N.D	3.3	3.3	66.4
	S9aug	N.D	17.1	6095.3	138639.8	144752.3	646.6	49.0	42.6	738.2	0.4	N.D	0.4	145490.8
	S9sept	11.6	30.6	21078.9	284749.8	305870.9	554.8	27.9	21.7	604.4	N.D	N.D	0.0	306475.3
S10	S10jul	N.D	7.4	429.2	13931.0	14367.6	6.1	28.0	52.5	86.5	N.D	3.3	3.3	14457.4
	S10aug	N.D	13.4	610.8	33556.3	34180.5	0.0	34.3	123.5	157.8	0.4	N.D	0.4	34338.7
	S10aept	12.8	6030.0	2183.7	46530.6	54757.1	8.1	17.0	94.1	119.2	N.D	N.D	0.0	54876.3

N.D., not detected

Table S2. Potential pathogenic bacteria detected in samples.

Genera	Human / Zoonotic pathogen	Species	Associated human diseases	Aerosolization	References
Acinetobacter spp.	Zoonotic	*A. baumannii*<br>*A. junii**<br>*A. johnsonii**	*Acinetobacter* infection		[1]
Aeromonas spp.	Zoonotic	*A. hydrophila,*	Gastroenteritis		[2]
Chromobacterium spp.	Human	*C. violaceum*	Chromobacteriosis		[3]
Citrobacter spp.	Zoonotic	*C. freundii**	Sepsis with brain abscess		[4]
Corynebacterium spp.	Zoonotic	*C. diphtheriae*	Diphtheria	Yes	[5]
Cutibacterium spp.	Human	*C. acnes**	Inflammation		[6]
Herbaspirillum spp.	Human	*H. huttiense**	Bacteremia		[7]
Legionella spp.	Human	*L. pneumophila*	Legionnaires' disease	Yes	[8]
Mycobacterium spp.	Zoonotic	*M. avium* complex group	Non-tuberculous mycobacterial (NTM) infections	Yes	[9,10]
Micrococcus spp.	Zoonotic	*M. flavus**	Endocariditis		[11]
Parachlamydia spp.	Zoonotic	*P. acanthamoeba**	Pneumonia	Yes	[12]
Plesiomonas spp.	Zoonotic	*P. shigelloides**	Gastroenteritis		[13]
Pseudomonas spp.	zoonotic	*P. aeruginosa*	*Pseudomonas* infection	Yes	[14]
Rhodococcus spp.	Zoonotic	*R. equi*	Pneumonia	Yes	[15]
Vibrio spp.	Zoonotic	*V. cholera**<br>*V. vulnificus**	Cholera disease<br>Gastroenteritis	Yes	[16,17]

*: bacterial species identified by 16s rRNA sequencing.

References

1. Joly-Guillou, M.L. Clinical impact and pathogenicity of Acinetobacter. *Clin. Microbiol. Infect.* **2005**, *11*, 868–873. https://doi.org/10.1111/j.1469-0691.2005.01227.x.
2. Fernández-Bravo, A.; Figueras, M.J. An Update on the Genus Aeromonas: Taxonomy, Epidemiology, and Pathogenicity. *Microorganisms* **2020**, *8*, 129. https://doi.org/10.3390/microorganisms8010129.
3. Soares, R.L.; Dias Neto, N.B.; Guizelini, C.C.; Araújo, M.A.; Leal, C.R.B.; Möck, T.B.M.; Ramos, C.A.N. Chromobacteriosis (Chromobacterium violaceum) in a calf from Brazil–case report. *Arq. Bras. Med. Veterinária Zootec.* **2019**, *71*, 1929–1933. https://doi.org/10.1590/1678-4162-11063.
4. Chen, X.-B.; Zhou, Y.-X.; Feng, Y. Citrobacter freundii induces sepsis with new-onset status seizure in an adult: A case report and literature review. *Med. (Baltim.)* **2023**, *102*, e32549. https://doi.org/10.1097/MD.0000000000032549.
5. World Health Organization. *Diphtheria*; World Health Organization: Geneva, Switzerland, 2024.
6. Mayslich, C.; Grange, P.A.; Dupin, N. Cutibacterium acnes as an Opportunistic Pathogen: An Update of Its Virulence-Associated Factors. *Microorganisms* **2021**, *9*, 303. https://doi.org/10.3390/microorganisms9020303.
7. Ruiz de Villa, A.; Alok, A.; Oyetoran, A.E.; Fabara, S.P. Septic Shock and Bacteremia Secondary to Herbaspirillum huttiense: A Case Report and Review of Literature. *Curēus (Palo Alto CA)* **2023**, *15*, e36155. https://doi.org/10.7759/cureus.36155.
8. Newton, H.J.; Ang, D.K.Y.; van Driel, I.R.; Hartland, E.L. Molecular Pathogenesis of Infections Caused by Legionella pneumophila. *Clin. Microbiol. Rev.* **2010**, *23*, 274–298. https://doi.org/10.1128/CMR.00052-09.
9. Balagué, N.; Uçkay, I.; Vostrel, P.; Hinrikson, H.; Van Aaken, I.; Beaulieu, J.Y. Non-tuberculous mycobacterial infections of the hand. *Chir. Main* **2015**, *34*, 18–23. https://doi.org/10.1016/j.main.2014.12.004.
10. Zhang, Z.X.; Cherng, B.P.Z.; Sng, L.-H.; Tan, Y.E. Clinical and microbiological characteristics of non-tuberculous mycobacteria diseases in Singapore with a focus on pulmonary disease, 2012-2016. *BMC Infect. Dis.* **2019**, *19*, 436. https://doi.org/10.1186/s12879-019-3909-3.
11. Ianniello, N.M.; Andrade, D.C.; Ivancic, S.; Eckardt, P.A.; Lemos Ramirez, J.C. Native valve infective endocarditis due to Micrococcus luteus in a non-Hodgkin's lymphoma patient. *IDCases* **2019**, *18*, e00657. https://doi.org/10.1016/j.idcr.2019.e00657.
12. Greub, G. Parachlamydia acanthamoebae, an emerging agent of pneumonia. *Clin. Microbiol. Infect.* **2009**, *15*, 18–28. https://doi.org/10.1111/j.1469-0691.2008.02633.x.
13. Escobar, J.C.; Bhavnani, D.; Trueba, G.; Ponce, K.; Cevallos, W.; Eisenberg, J. Plesiomonas shigelloides infection, Ecuador, 2004-2008. *Emerg. Infect. Dis.* **2012**, *18*, 322–324. https://doi.org/10.3201/eid1802.110562.
14. Qin, S.; Xiao, W.; Zhou, C.; Pu, Q.; Deng, X.; Lan, L.; Liang, H.; Song, X.; Wu, M. Pseudomonas aeruginosa: Pathogenesis, virulence factors, antibiotic resistance, interaction with host, technology advances and emerging therapeutics. *Signal Transduct. Target. Ther.* **2022**, *7*, 199. https://doi.org/10.1038/s41392-022-01056-1.
15. Roberts-Thomson, A.; Barnes, A.; Fielder, D.S.; Lester, R.J.G.; Adlard, R.D. Aerosol dispersal of the fish pathogen, Amyloodinium ocellatum. *Aquaculture* **2006**, *257*, 118–123. https://doi.org/10.1016/j.aquaculture.2006.02.058.
16. Jones, M.K.; Oliver, J.D. Vibrio vulnificus: Disease and Pathogenesis. *Infect. Immun.* **2009**, *77*, 1723–1733. https://doi.org/10.1128/IAI.01046-08.
17. Vanden Broeck, D.; Horvath, C.; De Wolf, M.J.S. Vibrio cholerae: Cholera toxin. *Int. J. Biochem. Cell Biol.* **2007**, *39*, 1771–1775. https://doi.org/10.1016/j.biocel.2007.07.005.

.
